# Cervical Cerclage Performed at 24–32+0 Weeks in Twin Pregnancies: A Multicenter Descriptive Cohort Study

**DOI:** 10.3390/medicina62091704

**Published:** 2026-09-05

**Authors:** Yuki Gen, Oyoung Kim, Inhye Shin, Yun Sung Jo, Jiyun Hong, Yiyoon Kang, Kyung Yoo, In Yang Park, Hyun Sun Ko, Subeen Hong

**Affiliations:** 1 Department of Obstetrics and Gynecology, St. Vincent’s Hospital, College of Medicine, The Catholic University of Korea, Suwon 16247, Republic of Korea; misuyuki@naver.com (Y.G.);; 2 Department of Obstetrics and Gynecology, Yeouido St. Mary’s Hospital, College of Medicine, The Catholic University of Korea, Seoul 07345, Republic of Korea; 3 Department of Obstetrics and Gynecology, Seoul St. Mary’s Hospital, College of Medicine, The Catholic University of Korea, 222 Banpo-daero, Seocho-gu, Seoul 06591, Republic of Korea; cindykyungyoo@gmail.com (K.Y.); mongkoko@catholic.ac.kr (H.S.K.)

**Keywords:** cervical cerclage at 24–32+0 weeks, twin pregnancy, gestational age, perinatal outcome, neonatal outcome, descriptive cohort

## Abstract

*Background and Objectives*: Twin pregnancies carry a high risk of preterm birth, and cerclage for a short or dilated cervix is typically placed before 24 weeks. Its use at or after 24 weeks is less well characterized in twins. *Materials and Methods*: We describe the perinatal course of twin pregnancies that underwent cervical cerclage at 24–32+0 weeks at three university hospitals in South Korea between 2015 and 2024, with pregnancies treated before 24 weeks as a descriptive reference. Entry into the late group required remaining undelivered at 24 weeks without prior cerclage, so the groups do not share a common baseline and were not compared. *Results*: Eighteen pregnancies received cerclage at 24–32+0 weeks (median 26.3 weeks), and 25 received it before 24 weeks. In the late group, delivery occurred at a median of 34.1 weeks (IQR 31.7–35.4) after a mean cerclage-to-delivery interval of 45.8 days (95% CI 34.7–56.9). No fetal, neonatal, or perinatal death occurred (0 of 36 fetuses in 18 pregnancies; 95% CI 0.0–17.6%, calculated on the 18 pregnancies). NICU admission was 83.3% (95% CI 62.3–93.8), and adverse neonatal morbidity was 55.6% (95% CI 34.2–75.0). *Conclusions*: No perinatal death was recorded in this descriptive series. Because no untreated control group was available, these findings do not establish efficacy but provide a real-world basis for future prospective controlled trials.

## 1. Introduction

Twin pregnancies carry a much higher risk of preterm birth than singleton pregnancies. Approximately 60% of twins are born before 37 weeks and 12% before 34 weeks, and preterm birth is a leading cause of neonatal morbidity and mortality in twins [1]. A short cervix in the second trimester is one of the strongest predictors of preterm birth in twins [2]. Cervical insufficiency and lower-genital-tract inflammation are central mechanisms of spontaneous preterm birth [3]. However, the optimal management of a short cervix in twin pregnancies remains uncertain.

Cervical cerclage was traditionally considered a contraindication in twin pregnancies with a short cervix. Meta-analyses of early randomized trials found no reduction in preterm birth and a possible increase [4,5]. Consequently, the American College of Obstetricians and Gynecologists (ACOG) and the Society for Maternal-Fetal Medicine (SMFM) do not recommend cerclage for a short cervix alone in twins [6,7]. However, these trials were small and pooled patients with heterogeneous indications, limiting the strength of their conclusions.

More recent studies have shifted the question from whether to use cerclage to identifying which patients benefit. Cerclage appears most useful in the highest-risk women, particularly those with a very short cervix (below 15 mm) or with cervical dilation on examination [8,9]. These benefits are largely derived from observational studies, and the overall quality of evidence is low [9]. Most of this evidence pertains to cerclage placed before 24 weeks.

Although cerclage placed at or after 24 weeks in twin pregnancies is encountered in real-world practice, evidence supporting its clinical course and outcomes in twin pregnancies remains sparse. Therefore, we describe the clinical characteristics and perinatal course of twin pregnancies that underwent cerclage at 24–32+0 weeks across three academic medical centers. To provide context, pregnancies treated before 24 weeks at the same three centers are presented as a descriptive reference and not as a comparison group. They were treated in the same period, but treatment in the same period does not give the two groups a common baseline, because a pregnancy could reach the late group only by remaining undelivered at 24 weeks without having already received cerclage.

## 2. Materials and Methods

### 2.1. Study Design and Participants

This multicenter retrospective study included women with twin pregnancies who underwent cervical cerclage at three hospitals affiliated with the College of Medicine, The Catholic University of Korea (Seoul St. Mary’s Hospital, Yeouido St. Mary’s Hospital, and St. Vincent’s Hospital) from January 2015 to December 2024. Multiple pregnancies delivered at these hospitals during the study period were identified from the institutional clinical data warehouse. Higher-order multiples were excluded, and every consecutive twin pregnancy that underwent cervical cerclage was included. No woman contributed more than one pregnancy. Pregnancies were divided into two groups based on gestational age at cerclage: an early group (<24 weeks) and a late group (24–32+0 weeks). Both boundaries were set before data extraction. We used 24+0 weeks as the lower boundary because the evidence for cerclage in twins does not extend past that point. ACOG confines both ultrasound-indicated and physical examination-indicated cerclage to before 24 weeks and does not recommend cerclage for a short cervix in twin pregnancies [6], and the randomized trial of physical examination-indicated cerclage in twins enrolled women only up to 23+6 weeks [8]. Above 24 weeks the guidance is inconsistent. The Royal College of Obstetricians and Gynaecologists (RCOG) extends rescue cerclage to 27+6 weeks, the International Federation of Gynecology and Obstetrics sets no upper limit [10,11], and a comparative review of twenty guidelines from ten societies found timing at or after 24 weeks to be one point of divergence [12]. Recent series of cerclages placed at or after 24 weeks have used comparable starting points, and placement as late as 30 weeks has been reported [13,14,15]. We therefore had to set an upper boundary rather than adopt one, and we anchored it to neonatal risk. Birth before 32 weeks covers the extremely preterm and very preterm categories, which account for about 15% of preterm births and carry the greatest need for neonatal intensive care [16]. This upper boundary was a screening rule and not a description of practice, since the latest cerclage in the cohort was placed at 30+4 weeks. We kept it at 32+0 weeks because a boundary redrawn after seeing the data would be defined by the data. The study was approved by the Institutional Review Board (approval number XC26RNDI0005), and the requirement for informed consent was waived due to its retrospective design.

Cerclage was placed using the McDonald technique in all cases. When the membranes were bulging, they were reduced with the Trendelenburg position and sponge forceps before the suture was placed. Before 24 weeks, the indication for cerclage followed conventional, internationally used criteria. These were a history of cervical insufficiency or prior early spontaneous preterm birth (history-indicated), a transvaginal cervical length below 2.5 cm without active preterm labor (ultrasound-indicated), or cervical dilatation with or without bulging membranes on examination (physical examination-indicated). At or after 24 weeks, no formal guideline or uniform institutional protocol governed cerclage placement, and the decision was individualized by the attending physician. Cerclage was offered to women whose cervix was markedly short or shortened further under observation and to women who presented with cervical dilatation and bulging membranes. No written numeric threshold was in force, but in practice cerclage was considered at a transvaginal cervical length of 2.0 cm or less, and all 18 procedures in the late group met that threshold, including the four in which the cervix was already dilated and the closed length was 0 cm. Vaginal progesterone was generally recommended for a short cervix but was not a prerequisite for cerclage. In the late group it was started before cerclage in 5 of the 18 women, on the day of cerclage in 1, and after cerclage in 1. Two women received another formulation only, and 9 of the 18 received no progesterone of any formulation. Inpatient observation was not mandatory, and in the late group, the cerclage followed admission by a median of 2 days (range 0 to 27). Three of the 9 women who received no progesterone underwent cerclage on the day of admission. No numeric definition of progressive shortening was applied. Consistent with this, the pre-cerclage cervical length in the late group was very short (median 0.71 cm, Table 1), ranging from a closed but extremely short cervix to overt cervical dilatation with prolapsing membranes. Before cerclage, each woman was assessed for the findings that define clinical chorioamnionitis in Section 2.2, and cerclage was withheld when it was suspected. Maternal C-reactive protein (CRP) and white blood cell (WBC) count supported this assessment and were within or near normal limits in most women (Table 1), with a median CRP of 0.26 mg/dL and a mean WBC count of 9159/µL. Both are routine preoperative tests and were obtained before cerclage and before any antibiotic was given. Antibiotics were given with the procedure as periprocedural prophylaxis and not as treatment for suspected infection, since none of the women had clinical signs of chorioamnionitis at the time of the procedure. Amniocentesis was not part of the routine work-up, so amniotic fluid culture and inflammatory markers were not available, and subclinical intra-amniotic infection therefore cannot be ruled out. Cerclage was not performed in the presence of active preterm labor, clinical chorioamnionitis, ruptured membranes, significant vaginal bleeding, or a known lethal fetal anomaly. Significant vaginal bleeding was assessed clinically and was taken to mean bleeding suspected to arise from a placental cause, such as abruption or placenta previa. Light bleeding attributable to cervical change was not treated as a contraindication, because it is common when the cervix dilates. No volume-based or numeric threshold was applied. Uterine contractions were identified on cardiotocography and cervical change on repeat transvaginal ultrasound. Active preterm labor required regular contractions at intervals of less than five minutes with severe labor pain, weighed alongside progressive cervical shortening or dilatation, so irregular contractions in a woman without symptoms did not preclude cerclage. Management was individualized, so the period of inpatient observation before the decision differed between women. The suture was removed once contractions became regular at intervals of two to three minutes with labor pain, and a repeat ultrasound showed appreciable cervical shortening. In the absence of labor, it was removed at cesarean delivery. The suture material and the type of anesthesia were not recorded as structured data and cannot be reported.

Adjunctive treatment was not standardized. Progesterone was given as a vaginal micronized 200 mg capsule, as an oral micronized capsule, or as an intramuscular depot. Every woman received a tocolytic, most often atosiban, nifedipine, or ritodrine, and the agent in use changed over the study period. Tocolytics were given routinely around the procedure to prevent the uterine irritability that cerclage can provoke and not only to arrest active labor. Antibiotics were given at cerclage in 40 of the 43 women, and neither the agent nor the duration was recorded. Antenatal corticosteroids were given when preterm birth was anticipated. Magnesium sulfate was not given at cerclage, and the two women who received it were given it close to delivery for fetal neuroprotection. There was no fixed length of stay after cerclage. Supplementary Table S9 reports the cointerventions in each group.

### 2.2. Data Collection and Definitions

Data were obtained from the electronic medical records of three hospitals and included maternal characteristics, laboratory and ultrasound findings, operative records, and delivery and neonatal outcomes. Baseline variables were maternal age, parity, pre-pregnancy body mass index (BMI), mode of conception (in vitro fertilization [IVF]), history of preterm birth, and chorionicity. Cerclage-related variables included gestational age at cerclage, indication for cerclage, cervical length, cervical dilatation or bulging of membranes, irregular uterine contractions and vaginal bleeding recorded as separate variables, CRP and WBC count at cerclage, adjunctive treatments (progesterone, antibiotics, tocolytics, antenatal corticosteroids, and magnesium sulfate), and the cerclage technique. The indication for cerclage was classified as history-indicated, ultrasound-indicated, or physical examination-indicated, consistent with a previous multicenter cerclage study from our institutions [17]. When more than one criterion was present, the most advanced cervical finding determined the category. Cervical dilatation on speculum examination took precedence over cervical length, and cervical length took precedence over obstetric history. The indication reflects the findings documented immediately before the procedure. It was not recorded prospectively as a study variable, and the category was assigned by retrospective review of the operative and clinical records. Cervical length was measured according to a standardized transvaginal ultrasound protocol with the bladder empty, and the shortest technically valid measurement was recorded. When the cervix was dilated and fully effaced, no closed canal remained, and the cervical length was recorded as 0 cm. This is a measured value and not a missing observation. Cervical dilatation and bulging membranes were assessed by speculum examination. Digital examination was not performed. The outcomes of interest were gestational age at delivery, the cerclage-to-delivery interval, delivery within 7 and 14 days of cerclage, preterm premature rupture of membranes (PPROM) within 48 h of cerclage, clinical chorioamnionitis, and preterm birth before 37, 34, 32, and 28 weeks. Clinical chorioamnionitis was defined as maternal fever (≥38.0 °C) accompanied by two or more of the following: uterine fundal tenderness, maternal tachycardia (>100 beats/min), fetal tachycardia (>160 beats/min), foul-smelling amniotic fluid, or maternal leukocytosis (>15,000 cells/mm^3^). Maternal and procedural outcomes were postpartum hemorrhage, red cell transfusion, genital tract laceration, uterine rupture, pelvic hematoma, puerperal fever, hysterectomy, maternal intensive care admission, and maternal death, taken from the diagnoses and procedures recorded at discharge using the same coding rules at all three centers. Postpartum hemorrhage was identified by its ICD-10 diagnosis code and not by a volume threshold. Blood loss at cesarean was taken from the operative record, and blood loss and transfusion are reported separately. Neonatal outcomes were birth weight, neonatal death, neonatal intensive care unit (NICU) admission, respiratory distress syndrome (RDS), intraventricular hemorrhage (IVH, any grade), bronchopulmonary dysplasia (BPD), necrotizing enterocolitis (NEC), hypoxic-ischemic encephalopathy (HIE), and neonatal sepsis, each taken from the discharge diagnosis recorded in the clinical data warehouse using the same coding rules at all three centers. For BPD, HIE, and sepsis, the discharge summary text was searched as well. The diagnoses were made by the attending neonatologists during the admission, using the criteria in routine use at the three centers, which follow those of the Korean Neonatal Network [18]. The three hospitals belong to one university system, share the electronic medical record, and applied the same definitions throughout the study period. RDS was clinical respiratory distress with a reticulogranular pattern and air bronchograms on chest radiography in an infant who needed supplemental oxygen or positive pressure support, together with surfactant replacement. IVH was germinal matrix or intraventricular hemorrhage on routine cranial ultrasonography, reported by the Papile classification [19]. The grade was not available for every affected neonate in the extracted dataset, so IVH is analyzed as hemorrhage of any grade. BPD was a continuing requirement for supplemental oxygen or respiratory support in an infant born before 32 weeks, assessed at 36 weeks postmenstrual age or at discharge, whichever came first [20]. NEC was feeding intolerance and abdominal distension with pneumatosis intestinalis, portal venous gas, or pneumoperitoneum on abdominal radiography, corresponding to modified Bell stage II or higher [21]. HIE was the diagnosis entered by the attending neonatologist, using the reference description of a depressed Apgar score with metabolic acidemia at birth followed by abnormal neurologic findings in the first days of life [22]. The stage was not available in the extracted dataset, so HIE covers encephalopathy of any severity. Neonatal sepsis was clinical and laboratory signs of systemic infection that prompted a full course of antibiotic therapy, with or without a pathogen isolated from blood or cerebrospinal fluid. Adverse neonatal morbidity was defined as the presence of one or more of RDS, IVH, BPD, NEC, HIE, or sepsis. These six conditions were combined because each is a recognized complication of preterm birth, and each was recorded in the same way at the three centers. A composite of this kind has been used in twin research [23]. The composite was exploratory and was not prespecified in a registered protocol, because this is a retrospective cohort and no protocol was registered before data extraction. It was defined in the statistical analysis plan for this study and applied identically to both groups. The composite does not include neonatal death as a component. We defined fetal death as delivery of a fetus with no sign of life, recorded as an Apgar score of 0 at both 1 and 5 min. We defined live birth as delivery with any sign of life. We defined neonatal death as death of a liveborn neonate before discharge from hospital. This is a discharge endpoint rather than a 7-day or 28-day endpoint, and no outcome after discharge was recorded. We defined perinatal death as fetal death or neonatal death. We did not use a separate category of stillbirth, because the gestational age threshold that separates stillbirth from earlier fetal loss differs between definitions. Every delivery with no sign of life is reported as a fetal death. Neonatal outcomes were analyzed in liveborn neonates only.

### 2.3. Statistical Analysis

Normality of continuous variables was assessed with the Shapiro-Wilk test. Continuous variables that were consistent with normality in both groups are presented as mean ± standard deviation (SD) with 95% confidence intervals derived from the t-distribution, and variables that departed from normality in either group are presented as median [interquartile range (IQR)]. On this basis, pre-pregnancy BMI, gestational age at cerclage, cervical length at cerclage, CRP at cerclage, gestational age at delivery, and birth weight are reported as median [IQR], and maternal age, WBC count at cerclage, and the cerclage-to-delivery interval are reported as mean ± SD. The length of the cerclage admission and blood loss at cesarean are reported as median (range). Supplementary Tables S5–S7 describe the physical examination-indicated subgroup, in which each group contains four pregnancies. In those three tables every continuous variable is given as the median, with the range and counts given without percentages or confidence intervals, because four observations cannot support them. Categorical variables are presented as numbers with percentages and 95% confidence intervals. For pregnancy-level variables the interval was calculated using the Wilson score method. Fetal and neonatal outcomes are reported per fetus or per neonate, and co-twins share a pregnancy, so those intervals were obtained from generalized estimating equations with the pregnancy as the cluster. The model used a binomial family with a logit link, an exchangeable working correlation, and robust sandwich standard errors, and there were 43 clusters at the level of the fetus and 41 among liveborn neonates. Intervals were formed on the logit scale and back-transformed, so they are not symmetric about the estimate. The group term is saturated, so each fitted probability equals the observed proportion, and no contrast between the groups was estimated. Where no event occurred in a group, or where every fetus had the outcome, the within-pair correlation is not estimable, so those estimates keep the Wilson score interval. This applies to neonatal death and to perinatal death in the late group and to live birth and survival to hospital discharge in that group in Supplementary Table S10. In each of these outcomes the result was the same for both fetuses of every pregnancy, so the interval was computed on the number of pregnancies rather than the number of fetuses, which is the wider and more conservative choice. Because this was a descriptive cohort without an expectant-management comparison group, no between-group significance testing was performed. Confidence intervals are provided to convey the precision of each estimate rather than to support inference about treatment effect. Descriptive analyses were performed with SAS version 9.4 (SAS Institute Inc., Cary, NC, USA).

## 3. Results

### 3.1. Study Population

**Figure 1:**
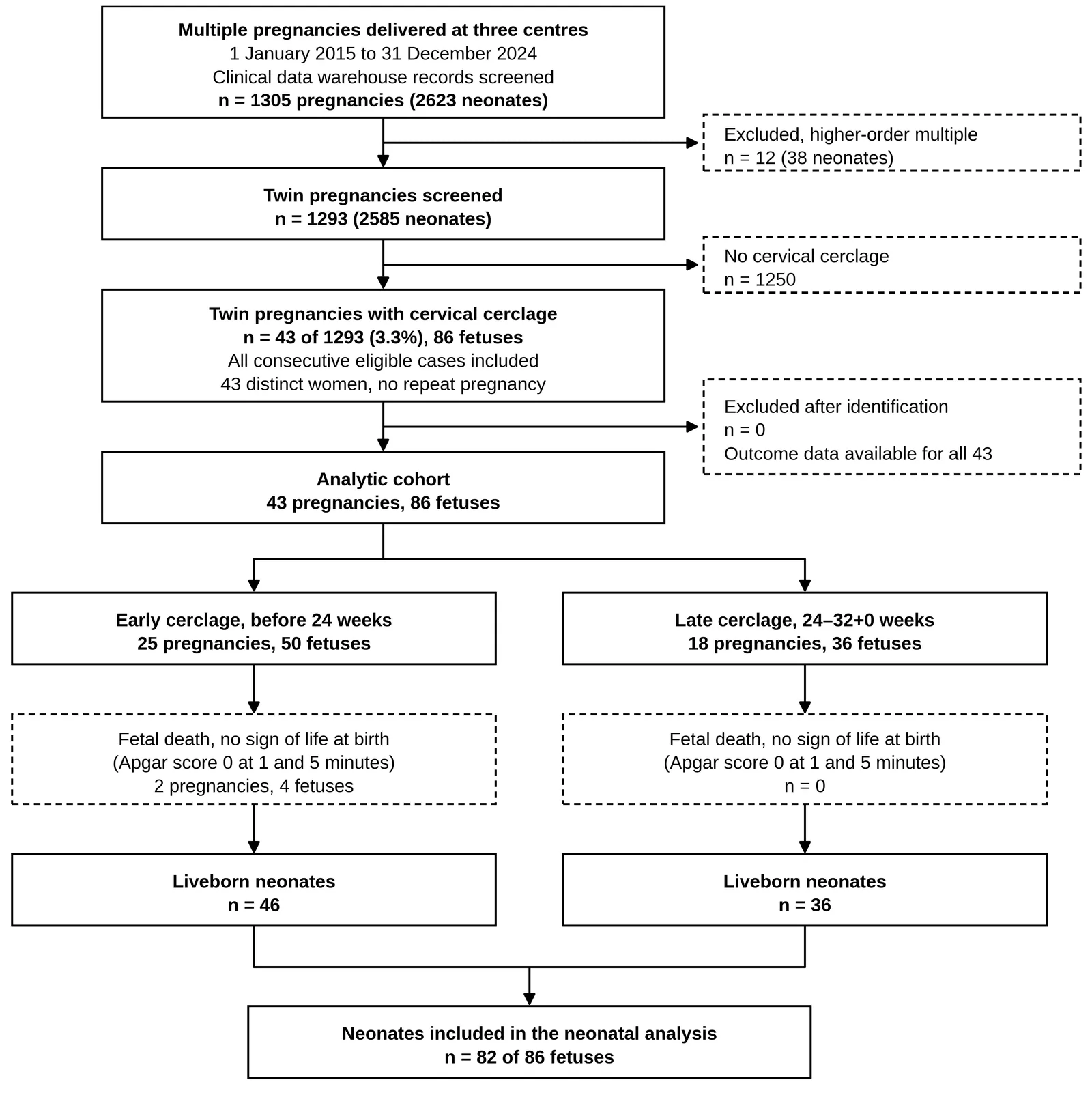
Flow of twin pregnancies through the study.

**Figure 2:**
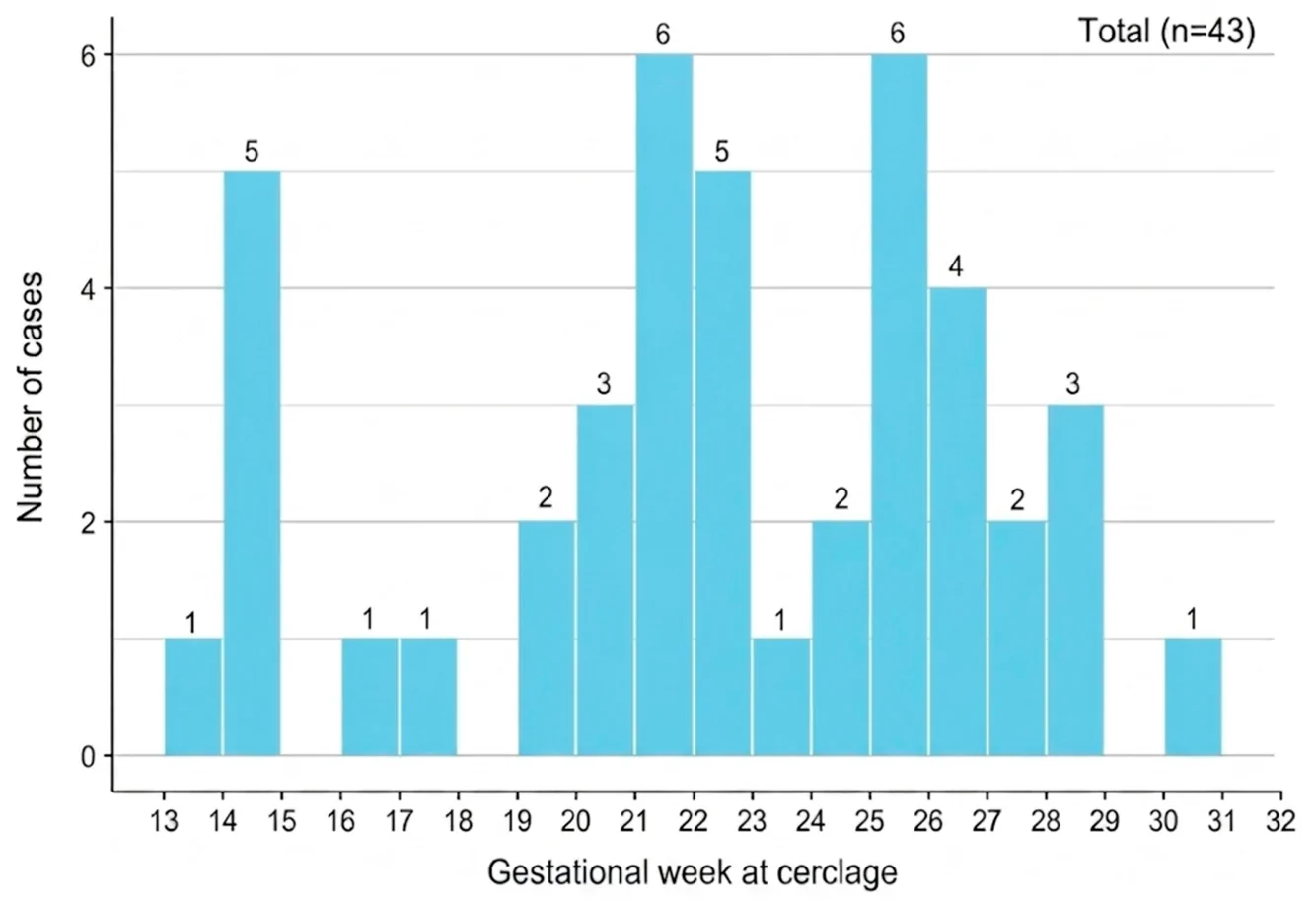
Distribution of gestational age at cerclage placement among the 43 twin pregnancies.

Between January 2015 and December 2024, 1305 multiple pregnancies were delivered at the three hospitals. Twelve were higher-order multiples, which left 1293 twin pregnancies. Cervical cerclage was performed in 43 of these 1293 pregnancies (3.3%), and all 43 were included. Cerclage was placed between 13+2 and 30+4 weeks, and delivery and neonatal outcome data were available for every pregnancy, so no pregnancy was excluded after identification. The 43 pregnancies produced 86 fetuses. Of these, 25 women received cerclage before 24 weeks and 18 received it at 24–32+0 weeks; hereafter, the early group and the late group. The flow of pregnancies through the study is shown in Figure 1. The distribution of gestational age at cerclage is shown in Figure 2, with cases clustering both before and after the 24-week threshold used to define the two groups. Within the late group, cerclage was placed at 24+0 to 25+6 weeks in 8 pregnancies (44.4%), at 26+0 to 27+6 weeks in 6 pregnancies (33.3%), and at or after 28+0 weeks in 4 pregnancies (22.2%). The earliest procedure in the late group was performed at 24+3 weeks and the latest at 30+4 weeks. The number of pregnancies in each stratum was small, so we describe them without comparing outcomes across strata. Case-level detail for all 18 pregnancies in the late group is given in Supplementary Table S8.

The three centers contributed unequally. One center performed 35 of the 43 cerclages (81.4%), a second 5 (11.6%), and a third 3 (7.0%), and relative to the twin pregnancies managed at each center, the cerclage rate was 4.4%, 1.8%, and 1.3%. Among the 18 cerclages in the late group, 12 (66.7%) were managed at the largest contributing center, 5 (27.8%) at the second, and 1 (5.6%) at the third. All five cerclages from the second center were in the late group and none in the early group, so center and the timing of cerclage are partly confounded. Centers are reported without attribution in Supplementary Tables S1 and S12. Regarding temporal distribution across the ten-year period, 27 cases occurred during 2015–2019 and 16 cases during 2020–2024. Of the 27 cerclages in the earlier period, 15 were early and 12 were late, and of the 16 in the later period, 10 were early and 6 were late, so the procedures in the late group were not concentrated in one part of the study period. Outcomes of the late group by calendar period are given in Supplementary Table S13. The distribution by center is shown in Supplementary Table S1, and the outcomes of the late group by center in Supplementary Table S12.

Two baseline variables were incomplete. Cervical length at cerclage was not measured in one early-group pregnancy, in which cerclage was history-indicated at 14+4 weeks. CRP at cerclage was not requested in four early-group pregnancies, three of them history-indicated procedures placed at 14 to 15 weeks. A blood count was drawn in all four, so the WBC figures rest on the full cohort. Every other variable in Table 1, Table 2 and Table 3 was recorded for all 43 pregnancies and all 82 liveborn neonates. No case was dropped from any analysis because of these gaps, and the number of observations behind each affected summary is given in the footnote to Table 1 and in Supplementary Table S14.

### 3.2. Baseline and Clinical Characteristics

Table 1 shows the baseline characteristics of both groups. In the late group the mean maternal age was 32.1 years, no woman had a history of preterm birth, the median gestational age at cerclage was 26.3 weeks, and the median cervical length at cerclage was 0.71 cm. Ultrasound-indicated cerclage accounted for 77.8% and physical examination-indicated cerclage for 22.2%, and no cerclage was history-indicated. In the early group the mean maternal age was 35.4 years, 24.0% had a history of preterm birth, the median gestational age at cerclage was 21.0 weeks, and the median cervical length was 1.20 cm, with history-indicated cerclage in 20.0%, ultrasound-indicated cerclage in 64.0%, and physical examination-indicated cerclage in 16.0%. All cerclages used the McDonald technique. The three indication categories are separated on the pre-cerclage findings. Cervical length was 3.2 to 4.0 cm in the four history-indicated pregnancies with a measurement, 0.2 to 2.3 cm in the 30 ultrasound-indicated pregnancies, and 0 cm in all eight physical examination-indicated pregnancies, in which the cervix was dilated and fully effaced so that no closed canal remained. Cervical dilatation or bulging membranes were present in all eight physical examination-indicated pregnancies and in none of the others. The groups differ in maternal age, history of preterm birth, gestational age at cerclage, cervical length, and indication. The two groups do not share a common baseline, because a pregnancy could enter the late group only by remaining undelivered at 24 weeks without having already received cerclage. The groups are shown side by side for description only and were not compared.

### 3.3. Indications for Cerclage in the Late Group

Eighteen twin pregnancies underwent cervical cerclage at 24–32+0 weeks, all by the McDonald technique. None were history-indicated. Fourteen (77.8%) were ultrasound-indicated and performed for cervical shortening detected on transvaginal ultrasound in the absence of active preterm labor (pre-cerclage cervical length 0.23–2.0 cm; median 1.0 cm) at a median gestational age of 26.7 weeks (range 25.0–30.6). The remaining four (22.2%) were physical examination-indicated cerclages, performed for cervical dilatation with bulging membranes (pre-cerclage cervical length 0 cm in all four) at a median gestational age of 25.4 weeks (range 24.4–27.0). Irregular uterine contractions were recorded in seven of the 18 pregnancies in the late group, five in the ultrasound-indicated subgroup and two in the physical examination-indicated subgroup. No woman in the late group had vaginal bleeding. The median gestational age at delivery was 32.9 weeks in the physical examination-indicated group and 34.2 weeks in the ultrasound-indicated group. With four pregnancies in the first of these, the figures are given for description only.

### 3.4. Pregnancy Outcomes

Table 2 shows the pregnancy outcomes of both groups. In the late group the median gestational age at delivery was 34.1 weeks (IQR 31.7–35.4), and the mean cerclage-to-delivery interval was 45.8 days (95% CI 34.7–56.9). The late group combines two clinically distinct indications, and the corresponding figures were 34.2 weeks and 46.4 days in the 14 ultrasound-indicated pregnancies and 32.9 weeks and a median of 55 days in the 4 physical examination-indicated pregnancies. The corresponding figures for the early group are given in Table 2. The interval is mechanically shorter when cerclage is placed closer to delivery, so it cannot be read across the two groups. Cerclage followed admission by a median of 2 days in the late group (IQR 0 to 5, range 0 to 27), by a median of 2.5 days in the ultrasound-indicated late subgroup, and by 0.5 days in the physical examination-indicated late subgroup. In the late group, delivery occurred within 7 days of the cerclage in 11.1% (95% CI 3.1–32.8), no additional deliveries occurred between 8 and 14 days, and 27.8% (95% CI 12.5–50.9) delivered within 28 days, so 13 of the 18 pregnancies continued for more than 4 weeks after the procedure. Preterm birth before 34 weeks was recorded in 38.9% (95% CI 20.3–61.4), before 32 weeks in 27.8% (95% CI 12.5–50.9), and before 28 weeks in 5.6% (95% CI 1.0–25.8). PPROM within 48 h occurred in 5.6% (95% CI 1.0–25.8), and no case of clinical chorioamnionitis occurred. The indication for delivery in the late group was preterm labor in 7 pregnancies, a planned cesarean in 4, recurrent cervical shortening in 2, PPROM in 2, fetal growth discordance in 2, and progressive cervical dilatation in 1. All 18 late-group pregnancies were delivered by cesarean. Supplementary Table S8 gives the indication for each case. Maternal and procedural safety outcomes are given in Supplementary Table S11. In one late pregnancy the cerclage did not maintain the pregnancy beyond the day of the procedure. A physical examination-indicated a cerclage placed at 25+3 weeks in a woman with a fully dilated cervix and bulging membranes was followed on the same day by PPROM and painful contractions, and she was delivered by cesarean that day. This is the only case of PPROM within 48 h in the late group and one of the two deliveries within 7 days. The record does not state whether the membranes ruptured during the procedure or after it, and the indication for delivery was recorded as preterm labor. PPROM was documented later in the pregnancy in a further 2 late-group pregnancies, at 26 and 66 days after cerclage. There was no genital tract laceration, uterine rupture, hysterectomy, maternal intensive care admission, or maternal death in either group. Postpartum hemorrhage occurred in 6 of the 18 late-group pregnancies, and the median blood loss at cesarean in that group was 500 mL. The early-group figures are given in Supplementary Table S11.

### 3.5. Neonatal Outcomes

The 43 pregnancies produced 86 fetuses, and we report the outcome of every one. Four fetuses were delivered with no sign of life and had an Apgar score of 0 at both 1 and 5 min. These four fetal deaths occurred in two early-group pregnancies delivered at 21.9 and 22.6 weeks, and in each of these pregnancies both fetuses died. No fetal death occurred in the late group. The remaining 82 fetuses were liveborn, 46 in the early group and 36 in the late group, so the neonatal analysis includes every liveborn neonate in the cohort, and no liveborn neonate was excluded. Four of the 46 liveborn neonates in the early group died before discharge, and these four deaths also occurred in two pregnancies, delivered at 24.0 and 25.9 weeks, in which both neonates died. The four deaths occurred on days 1, 5, 9, and 23 after birth, so all were within 28 days of birth. Perinatal death, defined as fetal death or death of a liveborn neonate before discharge, occurred in 8 of the 86 fetuses (9.3%, 95% CI 3.5–22.3), and all 8 occurred in the early group (16.0% of 50 fetuses, 95% CI 6.1–35.7). In total, 78 of the 86 fetuses survived to discharge (90.7%, 95% CI 77.7–96.5). Perinatal death occurred in 4 of the 25 early-group pregnancies and in none of the 18 late-group pregnancies, and each of those four pregnancies lost both fetuses. The complete outcome of all 86 fetuses, using fetal death, live birth, neonatal death, perinatal death, and survival to hospital discharge, is given in Supplementary Table S10.

Neonatal outcomes were assessed in the 82 liveborn neonates (46 early, 36 late). Table 3 displays the neonatal outcomes of the two groups. In the late group the median birth weight was 2.10 kg (IQR 1.56–2.35), no neonatal death occurred (0 of 36 neonates in 18 pregnancies; 95% CI 0.0–17.6, calculated on the 18 pregnancies rather than on the 36 neonates), NICU admission was 83.3% (95% CI 62.3–93.8), and adverse neonatal morbidity was 55.6% (95% CI 34.2–75.0). By indication, in the 28 neonates of the ultrasound-indicated late pregnancies, the median birth weight was 2.11 kg, NICU admission was 85.7%, and adverse neonatal morbidity was 57.1%. In the 8 neonates of the physical examination-indicated late pregnancies, the median birth weight was 1.93 kg, 6 of 8 were admitted to the NICU, and 4 of 8 had adverse neonatal morbidity, and these are given as counts because the denominator is too small for a percentage. Four neonatal deaths occurred in the early group (8.7%; 95% CI 3.4–20.3), and the remaining early-group figures are given in Table 3. Neonatal outcomes are also reported at the level of the pregnancy. Every pregnancy with a liveborn neonate had two liveborn neonates, so the pregnancy-level denominators are 23 in the early group, 18 in the late group, and 41 overall, while the neonate-level denominators are 46, 36, and 82. In the late group, at least one neonate was admitted to the NICU in 16 of the 18 pregnancies and both neonates in 14, and at least one neonate had adverse neonatal morbidity in 11 of the 18 pregnancies and both neonates in 9. A graded cranial ultrasound report was available for 8 of the 30 neonates with IVH. Grade 2 was recorded in 6 and grade 3 in 2, and no grade 4 occurred. All four neonates who died had at least one component of the composite, so no death falls outside it.

### 3.6. Subgroup Analyses by Indication for Cerclage

**Figure 3:**
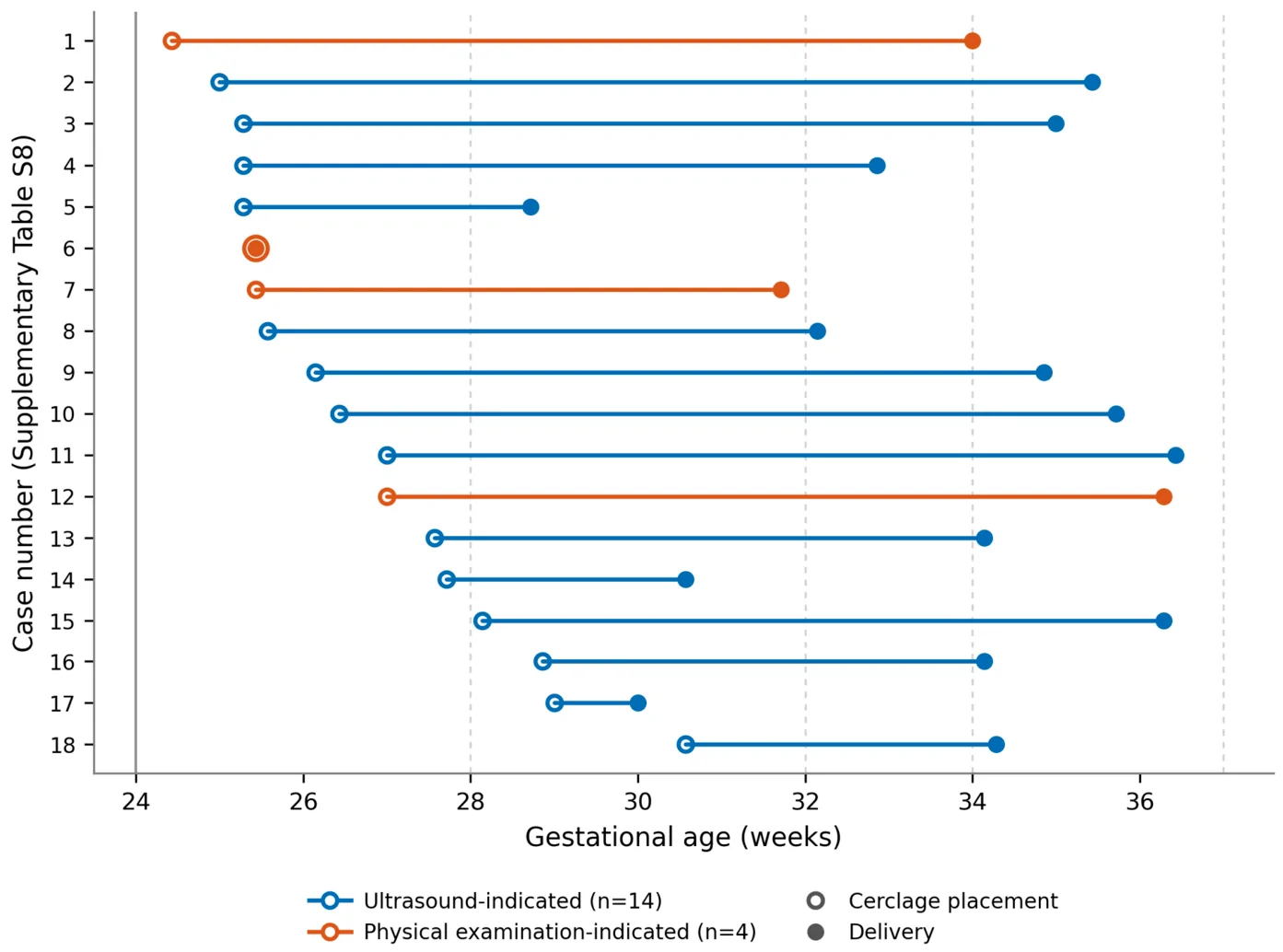
Gestational age at cerclage placement and at delivery for each of the 18 cerclages performed at 24–32+0 weeks. Each line is one pregnancy. The open circle marks cerclage placement, and the filled circle marks delivery. Case numbers are those of Supplementary Table S8. In Case 6 the two events fell on the same day, shown as a ring around the delivery marker. The solid vertical line marks 24 weeks, and the dashed lines mark 28, 32, 34, and 37 weeks.

The 18 cerclages in the late group were placed for two different clinical problems: 14 for cervical shortening found on ultrasound and 4 for cervical dilatation found on examination, and the course of each of the 18 pregnancies is shown in Figure 3. Among ultrasound-indicated cerclages (*n* = 30, 16 early and 14 late, 58 neonates) (Supplementary Tables S2–S4), the late group had a mean cerclage-to-delivery interval of 46.4 days and a median gestational age at delivery of 34.2 weeks. No neonatal death occurred among the liveborn neonates of the late group, NICU admission was 85.7%, and adverse neonatal morbidity was 57.1%. Two early-group neonates died. Preterm birth before 34, 32, and 28 weeks, and all early-group figures are given in Supplementary Tables S3 and S4.

In the physical examination-indicated subgroup (*n* = 8, 4 early and 4 late, 14 neonates) (Supplementary Tables S5–S7), the small number of cases precludes meaningful characterization. In the late group the median gestational age at delivery was 32.9 weeks, and the median cerclage-to-delivery interval was 55 days. No neonatal death occurred among the liveborn neonates of either group. The early-group figures are given in Supplementary Tables S6 and S7.

Given the small numbers, these subgroup figures are reported for completeness only and should not be interpreted as evidence of similarity or difference between the groups (Supplementary Tables S2–S7). Figure 3 shows the two indications separately for every late pregnancy, so the reader can see the individual courses rather than a pooled summary of a heterogeneous group.

## 4. Discussion

### 4.1. Principal Findings

In this multicenter retrospective series of 43 twin pregnancies that underwent cervical cerclage, we described the clinical course of 18 pregnancies treated at 24–32+0 weeks alongside 25 treated before 24 weeks. In the late group, delivery occurred at a median of 34.1 weeks (IQR 31.7–35.4), the mean interval from cerclage to delivery was 45.8 days, and no perinatal death was recorded (0 of 36 fetuses in 18 pregnancies; 95% CI 0.0–17.6%, calculated on the 18 pregnancies). NICU admission was 83.3%, and adverse neonatal morbidity was 55.6%. These figures describe the observed course of a selected cohort.

### 4.2. Current Guidelines and the Shift in Evidence

Current guidelines, including the 2024 SMFM Consult Series #70 and ACOG, do not recommend cerclage for a short cervix in twin pregnancies. This stance reflects older trials where cerclage did not reduce, and potentially increased, preterm birth [6,7].

In the first randomized trial of physical examination-indicated cerclage in twins, Roman et al. found that cerclage, given with indomethacin and antibiotics, significantly reduced perinatal death from 77% to 18% and lengthened the interval from diagnosis to delivery from 2.9 to 8.3 weeks. This trial, which enrolled women with cervical dilation of 1 to 5 cm up to 23+6 weeks, was halted early due to high mortality in the no-cerclage group [8]. A 2023 meta-analysis of 18 studies (1465 twin pregnancies) further indicated that cerclage for a short or dilated cervix was associated with a reduced risk of preterm birth before 28 weeks (RR 0.54, 95% CI 0.43–0.67), fewer perinatal deaths (RR 0.38, 95% CI 0.25–0.60), fewer NICU admissions (RR 0.75, 95% CI 0.63–0.90), and an average extension of pregnancy by 2.3 weeks. This benefit was primarily observed in twins with a cervix shorter than 15 mm or with cervical dilation and largely derived from observational rather than randomized data [9]. An earlier meta-analysis of twin cerclage studies similarly identified benefits in the highest-risk subgroups [24]. A 2025 systematic review of ultrasound-indicated cerclage in twins reached a cautiously optimistic conclusion. While it noted benefits in cohort studies and in women with a cervix shorter than 15 mm, it found no reduction in preterm birth in the randomized trials, prompting the authors to call for adequately powered randomized trials [25]. Overall, current evidence favors cerclage in the highest-risk twin pregnancies, those with a very short cervix (<15 mm) or an already dilated cervix, rather than for a short cervix (15 to 25 mm) alone. However, none of this evidence pertains to cerclage placed at or after 24 weeks.

### 4.3. Cerclage at or After 24 Weeks

Singleton data are more informative for cerclage placed at or after 24 weeks, and guidelines have begun to reflect this. The 2022 RCOG guideline allows rescue cerclage up to 27+6 weeks [10]. A meta-analysis of singleton randomized trials found a nonsignificant 22% reduction in preterm birth with cerclage at 24 to 26 weeks. This effect is similar to that of ultrasound-indicated cerclage before 24 weeks, prompting the authors to call for further trials [26]. Recent studies of rescue cerclage at 24–28 weeks and at 26–27+6 weeks reported longer pregnancies, later delivery, and better neonatal outcomes than expectant management, with no increase in infection or membrane rupture [13,14]. An individual patient data meta-analysis of physical examination-indicated cerclage at or after 24 weeks in singletons found lower odds of birth before 34 weeks (20.0% vs. 100%, OR 0.011, 95% CI 0.0004–0.31) and before 28 weeks (20.0% vs. 86.7%, OR 0.018, 95% CI 0.0009–0.35) than in matched controls. That analysis rested on only five cerclage cases, and none was placed at or after 26+0 weeks, so even in singletons there is no evidence beyond 26 weeks [27]. These singleton results cannot be applied directly to twins. In twin pregnancies, cerclage at or after 24 weeks remains unstudied rather than shown to be ineffective.

Our cohort adds twin-specific descriptive data to this clinically challenging area. Among twin pregnancies undergoing cerclage at 24–32+0 weeks (median 26.3 weeks), the observed mean interval between cerclage placement and delivery was 45.8 days (95% CI 34.7–56.9), and the median gestational age at delivery was 34.1 weeks, with no perinatal death recorded in the late group. Because this is an observational series without an untreated control group, these findings cannot establish that cerclage prolonged pregnancy and should be viewed as hypothesis-generating, describing the observed course of the late group.

### 4.4. Observed Neonatal Course

In our cohort no perinatal death occurred in the late group, and delivery occurred at a median of 34.1 weeks. NICU admission in that group was 83.3%, the median birth weight was 2.10 kg, and adverse neonatal morbidity was 55.6%. The NICU admission figure may reflect the low admission threshold for preterm twins and differing admission criteria across centers rather than worse neonatal health. These figures describe the observed neonatal course of the late group. They are not adjusted, and the two groups do not share a common baseline, so they were not compared.

### 4.5. Methodological Considerations

Group assignment in this cohort was defined by gestational age at intervention. By definition, a pregnancy could enter the late group only if it was still undelivered at 24 weeks and had not already received cerclage, whereas pregnancies in the early group remained at risk of previable loss throughout that interval. This constitutes immortal time and survival bias, and it is expected to favor the late group for every outcome reported here. The absence of neonatal deaths in the late group therefore cannot be interpreted as evidence of better or equivalent survival. In addition, four fetuses in the early group were delivered with no sign of life before 23 weeks and are reported as fetal deaths, so the poorest outcomes of that group fall outside any neonatal analysis by definition. Supplementary Table S10 therefore reports the outcome of all 86 fetuses. We also describe the early group as a descriptive reference from the same period, and we do not treat treatment in the same period as a reason to compare the groups. For these reasons we present the two groups descriptively and make no comparative or causal claim, and we intentionally avoided any adjusted or matched analysis that would imply a causal interpretation this design cannot support.

### 4.6. Strengths and Limitations

Strengths include the multicenter design, one set of variable definitions applied across three hospitals, and a focus on the understudied question of cerclage at or after 24 weeks in twins.

Several limitations should be considered. First, the cohort was small, retrospective, and non-randomized, lacking a no-cerclage comparison group. The study was not designed or powered to test equivalence.

Second, the study spanned a 10-year period during which obstetric and neonatal care may have evolved, and perioperative management was not standardized across centers. Cervical length surveillance, progesterone use, antenatal corticosteroid practice, thresholds for neonatal resuscitation and NICU admission, and neonatal care itself may all have changed over the ten years, and none of the estimates reported here is adjusted for calendar time. The procedures in the late group were not concentrated in one part of the period, with 12 of the 18 in 2015 to 2019 and 6 in 2020 to 2024, and Supplementary Table S13 gives their outcomes by period. The later period contains only 6 pregnancies, and those cerclages were placed at an earlier gestational age and at a shorter cervix, so the two periods cannot be read as a change in outcome over time. Adjunctive treatment was chosen by the attending physician, and the antibiotic and the tocolytic in use changed over the ten years, so this study cannot separate the effect of cerclage from the effect of the treatments given alongside it.

Third, no written protocol governed the cerclage at or after 24 weeks, so the criteria were not standardized across the three centers, and we cannot document how they differed. Which women entered the late group therefore depended on the threshold of the attending obstetrician, and selection by indication cannot be removed from a cohort assembled in this way.

Fourth, the centers contributed unequally. One center provided 35 of the 43 pregnancies, and all five pregnancies from a second center were in the late group, so the center and the timing of the cerclage are partly confounded. The centers differ in ways that bear directly on the outcomes we report. NICU admission among late-group neonates was 22 of 24 at the largest center and 8 of 12 at the other two centers combined, a difference more likely to reflect admission thresholds than neonatal health, and adverse neonatal morbidity was 17 of 24 and 3 of 12. Supplementary Table S12 gives the outcomes of the late group by center, without confidence intervals, because the numbers per center are too small to support them. The three hospitals also belong to a single university system that shares one electronic medical record. The unequal contribution of the centers and this shared setting may restrict the generalizability of the findings, which describe practice at the contributing centers rather than practice where admission thresholds and perioperative care differ.

Fifth, we could not exclude subclinical intra-amniotic infection. Amniocentesis was not performed, so no amniotic fluid culture or inflammatory marker was available, and the clinical findings that informed the pre-procedure assessment were held as free text rather than as structured data.

Sixth, the composite of adverse neonatal morbidity combines conditions of very different severity, so a neonate with transient respiratory distress counts the same as a neonate with severe IVH. A composite of that kind is driven mainly by its commonest components and by gestational age, and it is sensitive to local practice, as the difference in recorded morbidity between centers shows. It should be read as a marker of whether any complication of prematurity was recorded and not as a measure of severity. The record also limits how finely the neonatal outcomes can be described. The grade of IVH was not recorded for every affected neonate, so hemorrhage is reported at any grade. NEC was not staged, BPD was not graded, and neonatal sepsis was not separated into culture-proven and clinically suspected disease. Neonatal morbidity was taken from the diagnoses recorded at discharge and was not re-adjudicated for this study. IVH at any grade includes germinal matrix hemorrhage, and the recorded diagnosis of HIE was not staged, so both are best read as recorded-diagnosis rates rather than validated case counts. Intraoperative membrane rupture, cervical laceration at the time of cerclage, anesthetic complications, and suture displacement were not recorded as structured variables and could not be reported. Rupture of membranes beyond 48 h after cerclage is not a structured variable either, and the figures we give for it were taken from the recorded clinical course, so they may be an undercount.

Finally, we assessed outcomes only up to the neonatal period and did not evaluate long-term childhood neurodevelopmental outcomes.

## 5. Conclusions

In this multicenter retrospective series of twin pregnancies undergoing cervical cerclage at 24–32+0 weeks, the observed mean interval between cerclage placement and delivery was 45.8 days, and the observed median gestational age at delivery was 34.1 weeks, with no fetal, neonatal, or perinatal death among the 36 fetuses in this group. These descriptive findings do not establish therapeutic efficacy, because no untreated control group was available, and the course these pregnancies would have taken without a cerclage is unknown. They describe what was observed after cerclage at 24–32+0 weeks in a small and highly selected group. These data provide a real-world descriptive foundation to inform future prospective controlled trials.

## Figures and Tables

**Table 1:** Baseline maternal and cerclage-related characteristics of twin pregnancies by timing of cerclage placement.

Characteristic	Late (24–32+0 Weeks) (*N* = 18)	Early (<24 Weeks) (*N* = 25)
Maternal age, years	32.1 ± 5.3 [29.5–34.7]	35.4 ± 3.2 [34.1–36.7]
Nulliparity, *n* (%)	16 (88.9) [67.2–96.9]	16 (64.0) [44.5–79.8]
Pre-pregnancy BMI, kg/m^2^	20.4 [19.1–22.7]	22.2 [20.2–25.9]
IVF conception, *n* (%)	8 (44.4) [24.6–66.3]	12 (48.0) [30.0–66.5]
History of preterm birth, *n* (%)	0 (0.0) [0.0–17.6]	6 (24.0) [11.5–43.4]
Chorionicity, *n* (%)		
Monochorionic	3 (16.7) [5.8–39.2]	4 (16.0) [6.4–34.7]
Dichorionic	15 (83.3) [60.8–94.2]	21 (84.0) [65.3–93.6]
GA at cerclage, weeks	26.3 [25.3–27.7]	21.0 [16.9–21.9]
Indication, *n* (%)		
History-indicated	0 (0.0) [0.0–17.6]	5 (20.0) [8.9–39.1]
Ultrasound-indicated	14 (77.8) [54.8–91.0]	16 (64.0) [44.5–79.8]
Physical examination-indicated	4 (22.2) [9.0–45.2]	4 (16.0) [6.4–34.7]
Cervical length at cerclage, cm	0.71 [0.23–1.20]	1.20 [0.55–1.78]
Cervical dilatation or bulging, *n* (%)	4 (22.2) [9.0–45.2]	4 (16.0) [6.4–34.7]
Irregular uterine contractions, *n* (%)	7 (38.9) [20.3–61.4]	5 (20.0) [8.9–39.1]
Vaginal bleeding, *n* (%)	0 (0.0) [0.0–17.6]	1 (4.0) [0.7–19.5]
CRP at cerclage, mg/dL	0.26 [0.11–0.34]	0.20 [0.14–0.49]
WBC at cerclage, /µL	9159 ± 1969 [8180–10,138]	9493 ± 1807 [8747–10,239]
Vaginal progesterone use, *n* (%)	7 (38.9) [20.3–61.4]	10 (40.0) [23.4–59.3]
Antibiotic use, *n* (%)	17 (94.4) [74.2–99.0]	23 (92.0) [75.0–97.8]

Data are mean ± SD, median [interquartile range], or *n* (%). Brackets contain the 95% confidence interval for means (t-distribution) and for proportions (Wilson score method) and the interquartile range for medians. Pre-pregnancy BMI, GA at cerclage, cervical length at cerclage, and CRP at cerclage departed from normality on the Shapiro-Wilk test and are presented as median [IQR]; maternal age and WBC at cerclage are presented as mean ± SD. No between-group significance testing was performed. The two groups do not share a common baseline, because a pregnancy could enter the late group only by remaining undelivered at 24 weeks without having already received cerclage. The groups are shown side by side for description only and were not compared. Cervical length at cerclage was available for 24 of the 25 early-group pregnancies, and C-reactive protein at cerclage for 21 of the 25. Every other entry in this table rests on all 25 early-group and all 18 late-group pregnancies. BMI, body mass index; IVF, in vitro fertilization; GA, gestational age; CRP, C-reactive protein; WBC, white blood cell count.

**Table 2:** Pregnancy outcomes by timing of cerclage placement.

Outcome	Late (24–32+0 Weeks) (*N* = 18)	Early (<24 Weeks) (*N* = 25)
GA at delivery, weeks	34.1 [31.7–35.4]	34.1 [29.3–36.4]
Cerclage-to-delivery interval, days	45.8 ± 22.3 [34.7–56.9]	91.2 ± 44.2 [73.0–109.4]
Delivery within 7 days, *n* (%)	2 (11.1) [3.1–32.8]	1 (4.0) [0.7–19.5]
Delivery within 14 days, *n* (%)	2 (11.1) [3.1–32.8]	1 (4.0) [0.7–19.5]
Delivery within 28 days, *n* (%)	5 (27.8) [12.5–50.9]	2 (8.0) [2.2–25.0]
PPROM within 48 h, *n* (%)	1 (5.6) [1.0–25.8]	0 (0.0) [0.0–13.3]
Clinical chorioamnionitis, *n* (%)	0 (0.0) [0.0–17.6]	1 (4.0) [0.7–19.5]
Preterm birth < 37 weeks, *n* (%)	18 (100.0) [82.4–100.0]	20 (80.0) [60.9–91.1]
Preterm birth < 34 weeks, *n* (%)	7 (38.9) [20.3–61.4]	12 (48.0) [30.0–66.5]
Preterm birth < 32 weeks, *n* (%)	5 (27.8) [12.5–50.9]	8 (32.0) [17.2–51.6]
Preterm birth < 28 weeks, *n* (%)	1 (5.6) [1.0–25.8]	6 (24.0) [11.5–43.4]

Data are mean ± SD, median [interquartile range], or *n* (%), with brackets as in Table 1. GA at delivery departed from normality on the Shapiro-Wilk test and is presented as median [IQR]; the cerclage-to-delivery interval is presented as mean ± SD. No between-group significance testing was performed. The two groups do not share a common baseline, because a pregnancy could enter the late group only by remaining undelivered at 24 weeks without having already received cerclage. The groups are shown side by side for description only and were not compared. PPROM, preterm premature rupture of membranes; GA, gestational age.

**Table 3:** Neonatal outcomes by timing of cerclage placement (per neonate).

Neonatal Outcome	Late (24–32+0 Weeks) (*N* = 36)	Early (<24 Weeks) (*N* = 46)
Birth weight, kg ^a^	2.10 [1.56–2.35]	2.09 [1.81–2.50]
Neonatal death, *n* (%) ^b^	0 (0.0) [0.0–17.6]	4 (8.7) [3.4–20.3]
NICU admission, *n* (%)	30 (83.3) [62.3–93.8]	30 (65.2) [46.4–80.2]
Respiratory distress syndrome, *n* (%)	14 (38.9) [20.7–60.8]	14 (30.4) [17.3–47.8]
Intraventricular hemorrhage, *n* (%)	11 (30.6) [15.2–52.0]	19 (41.3) [24.5–60.4]
Bronchopulmonary dysplasia, *n* (%)	5 (13.9) [4.4–36.2]	10 (21.7) [10.6–39.5]
Necrotizing enterocolitis, *n* (%)	1 (2.8) [0.4–16.9]	1 (2.2) [0.3–13.6]
Hypoxic-ischemic encephalopathy, *n* (%)	3 (8.3) [2.9–21.9]	3 (6.5) [2.2–17.7]
Neonatal sepsis, *n* (%)	4 (11.1) [4.5–24.8]	8 (17.4) [8.7–31.8]
Adverse neonatal morbidity, *n* (%)	20 (55.6) [34.2–75.0]	26 (56.5) [39.1–72.5]

Data are mean ± SD, median [interquartile range], or *n* (%) per neonate. Values in brackets are 95% confidence intervals from generalized estimating equations clustered on the pregnancy, unless stated otherwise. ^a^ Median [interquartile range]. Birth weight departed from normality on the Shapiro-Wilk test. ^b^ Wilson score interval, because the clustered model cannot be fitted when no neonatal death occurred in the late group. All four deaths in the cohort arose in two pregnancies in which both neonates died, so death was fully clustered within the pregnancy, and the interval was therefore computed on the 18 pregnancies rather than on the 36 neonates. No between-group significance testing was performed. The two groups do not share a common baseline, because a pregnancy could enter the late group only by remaining undelivered at 24 weeks without having already received cerclage. The groups are shown side by side for description only and were not compared. Four fetuses in the early group were delivered with no sign of life and are reported as fetal deaths, so the early group contributed 50 fetuses and 46 liveborn neonates. No liveborn neonate was excluded. The outcome of all 86 fetuses is given in Supplementary Table S10. Adverse neonatal morbidity was defined as one or more of respiratory distress syndrome, intraventricular hemorrhage, bronchopulmonary dysplasia, necrotizing enterocolitis, hypoxic-ischemic encephalopathy, or neonatal sepsis. It is an exploratory composite outcome and was not prespecified in a registered protocol. NICU, neonatal intensive care unit.

## Data Availability

The data presented in this study are available on request from the corresponding author. The data are not publicly available owing to privacy restrictions.

## Supplementary Materials

The following supporting information can be downloaded at: https://www.mdpi.com/article/10.3390/medicina62091704/s1, Table S1: Distribution of the 43 cerclage cases across the three participating centers; Table S2: Baseline and clinical characteristics of the ultrasound-indicated subgroup, by timing of cerclage placement; Table S3: Pregnancy outcomes in the ultrasound-indicated subgroup, by timing of cerclage placement; Table S4: Neonatal outcomes in the ultrasound-indicated subgroup, by timing of cerclage placement (per neonate); Table S5: Baseline and clinical characteristics of the physical examination-indicated subgroup, by timing of cerclage placement; Table S6: Pregnancy outcomes in the physical examination-indicated subgroup, by timing of cerclage placement; Table S7: Neonatal outcomes in the physical examination-indicated subgroup, by timing of cerclage placement (per neonate); Table S8: Case-level summary of the 18 cervical cerclages placed at 24–32+0 weeks; Table S9: Cointerventions during the cerclage admission, by timing of cerclage placement; Table S10: Outcome of all 86 fetuses, by timing of cerclage placement; Table S11: Maternal and procedural safety outcomes, by timing of cerclage placement; Table S12: Characteristics and outcomes of the late group (cerclage at 24–32+0 weeks), by center; Supplementary Table S13. Characteristics and outcomes of the late group (cerclage at 24–32+0 weeks), by calendar period; Table S14: Number of observations available for each variable reported in Table 1, Table 2 and Table 3.

## Abbreviations

ACOG	American College of Obstetricians and Gynecologists
BMI	body mass index
BPD	bronchopulmonary dysplasia
CI	confidence interval
CRP	C-reactive protein
HIE	hypoxic-ischemic encephalopathy
IQR	interquartile range
IVF	in vitro fertilization
IVH	intraventricular hemorrhage
NEC	necrotizing enterocolitis
NICU	neonatal intensive care unit
OR	odds ratio
PPROM	preterm premature rupture of membranes
RCOG	Royal College of Obstetricians and Gynaecologists
RDS	respiratory distress syndrome
RR	relative risk
SD	standard deviation
SMFM	Society for Maternal-Fetal Medicine
WBC	white blood cell
