# Supplementary material for: Cervical Cerclage Performed at 24–32+0 Weeks in Twin Pregnancies: A Multicenter Descriptive Cohort Study
Source: Medicina (Kaunas). 2026 Sep 5;62(9):1704. doi: 10.3390/medicina62091704 (PMC13609619; doi:10.3390/medicina62091704)
Supplement: Supplementary file 1 [file medicina-62-01704-s001.zip › medicina-4519617-supplementary.pdf]

Supplementary Materials

Supplementary Table S1. Distribution of the 43 cerclage cases across the three participating centers

**Center**	**Twin pregnancies screened, n**	**Cerclage cases, n (%)**	**Cerclage rate at center, %**	**Late**<br>**(24–32+0 weeks)**	**Early**<br>**(<24 weeks)**
Center A	281	5 (11.6)	1.8	5	0
Center B	789	35 (81.4)	4.4	12	23
Center C	223	3 (7.0)	1.3	1	2
Total	1,293	43 (100)	3.3	18	25

Data are n (%); percentages in the third column are of all 43 cerclage cases. Center labels are arbitrary and do not follow the order of the author affiliations. Twin pregnancies screened are all twin pregnancies identified in the clinical data warehouse at that center during the study period, and the center-specific rate is the number of cerclage cases divided by that number. The cerclage rate was 1.8% at Center A, 4.4% at Center B and 1.3% at Center C, as a proportion of the twin pregnancies managed there. The three denominators sum to the 1,293 twin pregnancies screened. Cases were more frequent in the first half of the study period (27 in 2015–2019 vs 16 in 2020–2024).

Supplementary Table S2. Baseline and clinical characteristics of the ultrasound-indicated subgroup, by timing of cerclage placement

Characteristic	Late (24–32+0 weeks) (N=14)	Early (<24 weeks) (N=16)
Maternal age, years	32.5 ± 4.6 [29.8–35.2]	35.6 ± 3.4 [33.8–37.4]
Nulliparity, n (%)	12 (85.7) [60.1–96.0]	13 (81.3) [57.0–93.4]
Pre-pregnancy BMI, kg/m²	20.4 [19.4–22.7]	20.7 [20.1–22.6]
IVF, n (%)	6 (42.9) [21.4–67.4]	9 (56.3) [33.2–76.9]
History of preterm birth, n (%)	0 (0.0) [0.0–21.5]	1 (6.3) [1.1–28.3]
Monochorionic twins, n (%)	1 (7.1) [1.3–31.5]	4 (25.0) [10.2–49.5]
GA at cerclage, weeks	26.7 [25.3–28.1]	21.6 [20.1–22.4]
Cervical length, cm	1.00 [0.60–1.20]	1.20 [0.90–1.50]
Cervical dilatation or bulging, n (%)	0 (0.0) [0.0–21.5]	0 (0.0) [0.0–19.4]
Irregular uterine contractions, n (%)	5 (35.7) [16.3–61.2]	4 (25.0) [10.2–49.5]
Vaginal bleeding, n (%)	0 (0.0) [0.0–21.5]	0 (0.0) [0.0–19.4]
CRP at cerclage, mg/dL	0.28 [0.11–0.34]	0.20 [0.10–0.49]
WBC at cerclage, /µL	9012 ± 2031 [7839–10185]	9641 ± 1724 [8722–10560]
Vaginal progesterone use, n (%)	6 (42.9) [21.4–67.4]	8 (50.0) [28.0–72.0]

Antibiotic use, n (%)	14 (100.0) [78.5–100.0]	15 (93.8) [71.7–98.9]
McDonald technique, n (%)	14 (100.0) [78.5–100.0]	16 (100.0) [80.6–100.0]

Data are mean ± SD, median [interquartile range], or n (%). Brackets contain the 95% confidence interval for means (t-distribution) and for proportions (Wilson score method), and the interquartile range for medians. Pre-pregnancy BMI, GA at cerclage, cervical length, and CRP at cerclage departed from normality on the Shapiro-Wilk test and are presented as median [IQR]; maternal age and WBC at cerclage are presented as mean ± SD. No between-group significance testing was performed. C-reactive protein at cerclage was available for 15 of the 16 early-group pregnancies. Every other entry in this table rests on all 16 early-group and all 14 late-group pregnancies. BMI, body mass index; IVF, in vitro fertilization; GA, gestational age; CRP, C-reactive protein; WBC, white blood cell count.

Supplementary Table S3. Pregnancy outcomes in the ultrasound-indicated subgroup, by timing of cerclage placement

**Outcome**	**Late (24–32+0 weeks) (N=14)**	**Early (<24 weeks) (N=16)**
GA at delivery, weeks	34.2 [32.1–35.4]	33.4 [28.4–36.5]
Cerclage-to-delivery interval, days	46.4 ± 20.6 [34.5–58.3]	80.2 ± 36.4 [60.8–99.6]
Delivery within 7 days, n (%)	1 (7.1) [1.3–31.5]	0 (0.0) [0.0–19.4]
Delivery within 14 days, n (%)	1 (7.1) [1.3–31.5]	0 (0.0) [0.0–19.4]
PPROM within 48 h, n (%)	0 (0.0) [0.0–21.5]	0 (0.0) [0.0–19.4]
Clinical chorioamnionitis, n (%)	0 (0.0) [0.0–21.5]	1 (6.3) [1.1–28.3]
Preterm birth <37 weeks, n (%)	14 (100.0) [78.5–100.0]	13 (81.3) [57.0–93.4]
Preterm birth <34 weeks, n (%)	5 (35.7) [16.3–61.2]	9 (56.3) [33.2–76.9]
Preterm birth <32 weeks, n (%)	3 (21.4) [7.6–47.6]	6 (37.5) [18.5–61.4]
Preterm birth <28 weeks, n (%)	0 (0.0) [0.0–21.5]	4 (25.0) [10.2–49.5]

Data are mean ± SD, median [interquartile range], or n (%), with brackets as in Supplementary Table S2. GA at delivery is presented as median [IQR]; the cerclage-to-delivery interval is presented as mean ± SD. No between-group significance testing was performed. GA, gestational age; PPROM, preterm premature rupture of membranes.

Supplementary Table S4. Neonatal outcomes in the ultrasound-indicated subgroup, by timing of cerclage placement (per neonate)

**Outcome**	**Late (24–32+0 weeks) (N=28)**	**Early (<24 weeks) (N=30)**
Birth weight, kg	2.11 [1.56–2.32]	2.06 [1.30–2.46]
Neonatal death, n (%)	0 (0.0) [0.0–21.5]	2 (6.7) [0.9–35.2]
NICU admission, n (%)	24 (85.7) [63.0–95.5]	21 (70.0) [47.1–86.0]
Respiratory distress syndrome, n (%)	11 (39.3) [18.7–64.5]	11 (36.7) [20.0–57.3]
Intraventricular hemorrhage, n (%)	9 (32.1) [15.2–55.6]	13 (43.3) [23.5–65.5]
Bronchopulmonary dysplasia, n (%)	2 (7.1) [1.0–37.0]	5 (16.7) [6.3–37.2]
Necrotizing enterocolitis, n (%)	1 (3.6) [0.5–20.8]	1 (3.3) [0.5–19.6]
Hypoxic-ischemic encephalopathy, n (%)	1 (3.6) [0.5–20.8]	1 (3.3) [0.5–19.6]
Neonatal sepsis, n (%)	2 (7.1) [1.9–23.4]	6 (20.0) [8.7–39.7]
Adverse neonatal morbidity, n (%)	16 (57.1) [33.4–78.0]	17 (56.7) [36.3–75.0]

Data are mean ± SD, median [interquartile range], or n (%) per neonate. Values in brackets are 95% confidence intervals from generalized estimating equations clustered on the pregnancy. Where no event occurred in a group, or where every neonate had the outcome, the clustered model is not estimable and the Wilson score interval is given. In that row the outcome was the same for both neonates of every pregnancy, so the interval was computed on the number of pregnancies rather than the number of neonates, which is 14 in the late group. Birth weight is presented as median [IQR]. No between-group significance testing was performed. Adverse neonatal morbidity was defined as one or more of respiratory distress syndrome, intraventricular hemorrhage, bronchopulmonary dysplasia, necrotizing enterocolitis, hypoxic-ischemic encephalopathy, or neonatal sepsis. It is an exploratory composite outcome and was not prespecified in a registered protocol. NICU, neonatal intensive care unit.

Supplementary Table S5. Baseline and clinical characteristics of the physical examination-indicated subgroup, by timing of cerclage placement.

Characteristic	Late (24–32+0 weeks) (N=4)	Early (<24 weeks) (N=4)
Maternal age, years	33 (19–38)	33 (29–39)
Nulliparity, n/N	4/4	3/4
Pre-pregnancy BMI, kg/m²	20.8 (18.4–25.1)	25.7 (18.9–33.2)
IVF, n/N	2/4	1/4
History of preterm birth, n/N	0/4	0/4
Monochorionic twins, n/N	2/4	0/4
GA at cerclage, weeks	25.4 (24.4–27.0)	20.9 (17.6–22.9)
Cervical length, cm	0.00 (0.00–0.00)	0.00 (0.00–0.00)
Cervical dilatation or bulging, n/N	4/4	4/4
Irregular uterine contractions, n/N	2/4	1/4
Vaginal bleeding, n/N	0/4	1/4
CRP at cerclage, mg/dL	0.12 (0.03–0.96)	0.43 (0.14–0.57)
WBC at cerclage, /µL	8795 (8570–12530)	8925 (8050–12270)
Vaginal progesterone use, n/N	1/4	1/4
Antibiotic use, n/N	3/4	4/4
McDonald technique, n/N	4/4	4/4

Data are counts or the median with the range in parentheses. Each group contains four pregnancies, so no percentages and no confidence intervals are given. Counts are the number affected out of the number in the group, and every continuous variable is given as the median with the range, including variables reported as mean ± SD elsewhere in the manuscript. This differs from the other supplementary tables and is intended to avoid conveying a precision that these numbers cannot support. No between-group significance testing was performed. Abbreviations as in Supplementary Table S2.

Supplementary Table S6. Pregnancy outcomes in the physical examination-indicated subgroup, by timing of cerclage placement

**Outcome**	**Late (24–32+0 weeks) (N=4)**	**Early (<24 weeks) (N=4)**
GA at delivery, weeks	32.9 (25.4–36.3)	34.4 (21.9–36.4)
Cerclage-to-delivery interval, days	55 (0–67)	88 (7–130)
Delivery within 7 days, n/N	1/4	1/4
Delivery within 14 days, n/N	1/4	1/4
PPROM within 48 h, n/N	1/4	0/4
Clinical chorioamnionitis, n/N	0/4	0/4
Preterm birth <37 weeks, n/N	4/4	4/4
Preterm birth <34 weeks, n/N	2/4	2/4
Preterm birth <32 weeks, n/N	2/4	1/4
Preterm birth <28 weeks, n/N	1/4	1/4

Data are counts or the median with the range in parentheses. Each group contains four pregnancies, so no percentages and no confidence intervals are given. Counts are the number affected out of the number in the group, and every continuous variable is given as the median with the range, including variables reported as mean ± SD elsewhere in the manuscript. This differs from the other supplementary tables and is intended to avoid conveying a precision that these numbers cannot support. No between-group significance testing was performed. Abbreviations as in Supplementary Table S3.

Supplementary Table S7. Neonatal outcomes in the physical examination-indicated subgroup, by timing of cerclage placement (per neonate)

**Outcome**	**Late**<br>**(24–32+0 weeks)**<br>**(N=8)**	**Early**<br>**(<24 weeks)**<br>**(N=6)**
Birth weight, kg	1.93 (0.84–2.58)	2.06 (1.97–2.71)
Neonatal death, n/N	0/8	0/6
NICU admission, n/N	6/8	2/6
Respiratory distress syndrome, n/N	3/8	1/6
Intraventricular hemorrhage, n/N	2/8	2/6
Bronchopulmonary dysplasia, n/N	3/8	2/6
Necrotizing enterocolitis, n/N	0/8	0/6
Hypoxic-ischemic encephalopathy, n/N	2/8	0/6
Neonatal sepsis, n/N	2/8	0/6
Adverse neonatal morbidity, n/N	4/8	2/6

Data are counts or the median with the range in parentheses. The groups contain six and eight neonates, so no percentages and no confidence intervals are given. Counts are the number affected out of the number in the group, and every continuous variable is given as the median with the range, including variables reported as mean ± SD elsewhere in the manuscript. This differs from the other supplementary tables and is intended to avoid conveying a precision that these numbers cannot support. No between-group significance testing was performed. Adverse neonatal morbidity and abbreviations are defined as in Supplementary Table S4.

Supplementary Table S8. Case-level summary of the 18 cervical cerclages placed at 24–32+0 weeks

Case	GA at cerclage, wk+d	Indication	Cervical length, cm	Cervical dilatation or bulging	Irregular uterine contractions	GA at delivery, wk+d	Cerclage-to-delivery interval, days	Indication for delivery
1	24+3	Physical examination	0.0	Yes	No	34+0	67	Preterm labor
2	25+0	Ultrasound	1.8	No	Yes	35+3	73	Recurrent cervical shortening
3	25+2	Ultrasound	0.5	No	No	35+0	68	Planned cesarean
4	25+2	Ultrasound	0.7	No	No	32+6	53	Preterm labor
5	25+2	Ultrasound	0.23	No	No	28+5	24	Progressive cervical dilatation
6	25+3	Physical examination	0.0	Yes	Yes	25+3	0	Preterm labor
7	25+3	Physical examination	0.0	Yes	No	31+5	44	Preterm labor
8	25+4	Ultrasound	1.0	No	No	32+1	46	Fetal growth discordance
9	26+1	Ultrasound	1.2	No	Yes	34+6	61	Preterm labor
10	26+3	Ultrasound	1.5	No	No	35+5	65	Fetal growth discordance
11	27+0	Ultrasound	1.0	No	Yes	36+3	66	PPROM
12	27+0	Physical examination	0.0	Yes	Yes	36+2	65	Planned cesarean
13	27+4	Ultrasound	1.0	No	No	34+1	46	Recurrent cervical shortening
14	27+5	Ultrasound	0.5	No	No	30+4	20	Preterm labor
15	28+1	Ultrasound	1.2	No	No	36+2	57	Planned cesarean
16	28+6	Ultrasound	2.0	No	No	34+1	37	Planned cesarean
17	29+0	Ultrasound	0.72	No	Yes	30+0	7	Preterm labor
18	30+4	Ultrasound	0.6	No	Yes	34+2	26	PPROM

Cases are ordered by gestational age at cerclage. All 18 procedures used the McDonald technique. Indication was ultrasound in 14 cases and physical

examination in 4. Cervical length is given as 0.0 cm in the four physical examination-indicated cases, in which the cervix was dilated and no length could be measured. Where the operative note recorded the extent of cervical dilatation it is given in parentheses, and in the other two cases the note describes cervical dilatation with bulging membranes that was not measured. Irregular uterine contractions denotes contractions traced on cardiotocography at the time of cerclage in the absence of regular painful contractions. No woman in the late group had vaginal bleeding. No neonate in the late group died before discharge. GA, gestational age; wk+d, completed weeks plus days.

Supplementary Table S9. Cointerventions during the cerclage admission, by timing of cerclage placement

Cointervention	Late<br>(24–32+0 weeks)<br>(N=18)	Early<br>(<24 weeks)<br>(N=25)
Vaginal micronized progesterone 200 mg	7 (38.9) [20.3–61.4]	10 (40.0) [23.4–59.3]
Oral micronized progesterone	4 (22.2) [9.0–45.2]	3 (12.0) [4.2–30.0]
Intramuscular progesterone depot	2 (11.1) [3.1–32.8]	6 (24.0) [11.5–43.4]
Antibiotics at cerclage	17 (94.4) [74.2–99.0]	23 (92.0) [75.0–97.8]
Any tocolytic	18 (100.0) [82.4–100.0]	25 (100.0) [86.7–100.0]
Atosiban	16 (88.9) [67.2–96.9]	16 (64.0) [44.5–79.8]
Nifedipine	13 (72.2) [49.1–87.5]	12 (48.0) [30.0–66.5]
Ritodrine	10 (55.6) [33.7–75.4]	24 (96.0) [80.5–99.3]
Any antenatal corticosteroid	15 (83.3) [60.8–94.2]	19 (76.0) [56.6–88.5]
Dexamethasone	13 (72.2) [49.1–87.5]	15 (60.0) [40.7–76.6]
Betamethasone	5 (27.8) [12.5–50.9]	8 (32.0) [17.2–51.6]
Magnesium sulfate	1 (5.6) [1.0–25.8]	1 (4.0) [0.7–19.5]
Length of cerclage admission, days	28 (5–92)	17 (2–122)

Data are n (%) with 95% confidence intervals in brackets (Wilson score method), except for the length of admission, which is the median (range). Women could receive more than one tocolytic and more than one progesterone formulation, so the categories are not mutually exclusive. Tocolytics were given as routine perioperative prophylaxis, to prevent the uterine irritability that cerclage can provoke, and not only to arrest active labor. The agents were often

given in sequence during the same admission, one being replaced by another, so the individual tocolytic rows sum to more than the number of women and to more than 100%. Antibiotic use was recorded as given or not given, and neither the agent nor the duration was available. Magnesium sulfate was started close to delivery in both women, for fetal neuroprotection rather than as a periprocedural tocolytic. No between-group significance testing was performed.

Supplementary Table S10. Outcome of all 86 fetuses, by timing of cerclage placement

**Outcome**	**Late (24–32+0 weeks) (N=36)**	**Early (<24 weeks) (N=50)**
Fetal death	0 (0.0) [0.0–17.6]	4 (8.0) [2.0–26.9]
Live birth	36 (100.0) [82.4–100.0]	46 (92.0) [73.1–98.0]
Neonatal death	0 (0.0) [0.0–17.6]	4 (8.0) [2.0–26.9]
Perinatal death	0 (0.0) [0.0–17.6]	8 (16.0) [6.1–35.7]
Survival to hospital discharge	36 (100.0) [82.4–100.0]	42 (84.0) [64.3–93.9]

Data are n (%) of fetuses with 95% confidence intervals in brackets, obtained from generalized estimating equations clustered on the pregnancy. Where no event occurred in a group, or where every fetus had the outcome, the clustered model is not estimable and the Wilson score interval is given. In those rows the outcome was the same for both fetuses of every pregnancy, so the interval was computed on the number of pregnancies rather than the number of fetuses, which is 18 in the late group. This applies to the rows in which no death occurred and to the rows in which every fetus was liveborn and survived. The denominator is every fetus, which is 50 in the early group, 36 in the late group, and 86 overall. Fetal death was defined as delivery with no sign of life, recorded as an Apgar score of 0 at both 1 and 5 minutes. Neonatal death was death of a liveborn neonate before discharge, and the percentages for neonatal death in this table use the fetal denominator rather than the liveborn denominator used in Table 3. Perinatal death was fetal death or neonatal death. All four fetal deaths occurred before 23 weeks, in two early-group pregnancies delivered at 21.9 and 22.6 weeks. No fetus delivered at or after 23 weeks died before birth, so previable delivery and fetal death describe the same four fetuses in this cohort. No separate category of stillbirth was used. No between-group significance testing was performed.

Supplementary Table S11. Maternal and procedural safety outcomes, by timing of cerclage placement

**Outcome**	**Late (24–32+0 weeks) (N=18)**	**Early (<24 weeks) (N=25)**
PPROM within 48 hours of cerclage	1 (5.6) [1.0–25.8]	0 (0.0) [0.0–13.3]
PPROM documented later in pregnancy	2 (11.1) [3.1–32.8]	3 (12.0) [4.2–30.0]
Clinical chorioamnionitis	0 (0.0) [0.0–17.6]	1 (4.0) [0.7–19.5]
Postpartum hemorrhage	6 (33.3) [16.3–56.3]	7 (28.0) [14.3–47.6]
Red cell transfusion at cesarean	0 (0.0) [0.0–17.6]	2 (8.0) [2.2–25.0]
Genital tract laceration	0 (0.0) [0.0–17.6]	0 (0.0) [0.0–13.3]
Uterine rupture	0 (0.0) [0.0–17.6]	0 (0.0) [0.0–13.3]
Pelvic hematoma	0 (0.0) [0.0–17.6]	1 (4.0) [0.7–19.5]
Puerperal fever	1 (5.6) [1.0–25.8]	1 (4.0) [0.7–19.5]
Hysterectomy	0 (0.0) [0.0–17.6]	0 (0.0) [0.0–13.3]
Maternal intensive care admission	0 (0.0) [0.0–17.6]	0 (0.0) [0.0–13.3]
Maternal death	0 (0.0) [0.0–17.6]	0 (0.0) [0.0–13.3]
Blood loss at cesarean, mL	500 (150–1000)	500 (200–1000)

Data are n (%) of pregnancies with 95% confidence intervals in brackets (Wilson score method), except for blood loss at cesarean, which is the median (range) among the 34 women for whom it was recorded, 16 of the 18 pregnancies in the late group and 18 of the 25 in the early group. Postpartum hemorrhage is the diagnosis recorded at discharge and is not defined by a volume threshold. Preterm premature rupture of membranes within 48 hours of cerclage is a recorded variable. Rupture later in the pregnancy is not, and was ascertained from the recorded clinical course, so it captures only rupture that was documented there and may be an undercount. The row for rupture later in the pregnancy does not include the pregnancy with rupture within 48 hours, and rupture was documented at some point after cerclage in 3 of the 25 early-group and 3 of the 18 late-group pregnancies. Intraoperative membrane rupture, cervical laceration at the time of cerclage, anesthetic complications, and suture displacement were not recorded as structured variables and could not be reported. No between-group significance testing was performed.

Supplementary Table S12. Characteristics and outcomes of the late group (cerclage at 24–32+0 weeks), by center

Variable	Largest contributing center (N=12)	Other two centers combined (N=6)	Late group, total (N=18)
GA at cerclage, weeks, median (range)	25.5 (25.0–28.9)	27.0 (24.4–30.6)	26.3 (24.4–30.6)
Cervical length at cerclage, cm, median (range)	0.85 (0.00–2.00)	0.66 (0.00–1.20)	0.71 (0.00–2.00)
Ultrasound-indicated, n	10	4	14
Physical examination-indicated, n	2	2	4
GA at delivery, weeks, median (range)	33.5 (25.4–36.3)	34.6 (30.0–36.4)	34.1 (25.4–36.4)
Cerclage-to-delivery interval, days, median (range)	46 (0–73)	63 (7–67)	50 (0–73)
Liveborn neonates, n	24	12	36
NICU admission, n	22	8	30
Adverse neonatal morbidity, n	17	3	20

Data are counts, or the median with the range in parentheses. One center contributed 35 of the 43 cerclages and 12 of the 18 cerclages in the late group, and the two smaller centers are pooled here because one of them contributed a single cerclage in the late group, which could not be reported separately without identifying that pregnancy. No confidence intervals are given, because the numbers are too small to support them. No fetal, neonatal, or perinatal death occurred in either column. Adverse neonatal morbidity is defined as in Table 3. GA, gestational age; NICU, neonatal intensive care unit.

Supplementary Table S13. Characteristics and outcomes of the late group (cerclage at 24–32+0 weeks), by calendar period

Variable	2015–2019 (N=12)	2020–2024 (N=6)	Late group, total (N=18)
GA at cerclage, weeks, median (range)	27.3 (25.0–30.6)	25.3 (24.4–25.6)	26.3 (24.4–30.6)
Cervical length at cerclage, cm, median (range)	1.00 (0.00–2.00)	0.37 (0.00–1.00)	0.71 (0.00–2.00)
Ultrasound-indicated, n	10	4	14
Physical examination-indicated, n	2	2	4
Vaginal progesterone use, n	4	3	7
Any antenatal corticosteroid, n	9	6	15
GA at delivery, weeks, median (range)	34.6 (25.4–36.4)	32.5 (28.7–35.0)	34.1 (25.4–36.4)
Cerclage-to-delivery interval, days, median (range)	52 (0–73)	50 (24–68)	50 (0–73)
Preterm birth <34 weeks, n	3	4	7
Preterm birth <32 weeks, n	3	2	5
Preterm birth <28 weeks, n	1	0	1
Liveborn neonates, n	24	12	36
NICU admission, n	19	11	30
Adverse neonatal morbidity, n	14	6	20

Data are counts, or the median with the range in parentheses. The period is that of delivery. No confidence intervals and no between-period testing are given, because 6 pregnancies cannot support them. The later period contains fewer pregnancies and those cerclages were placed earlier and at a shorter cervix, so the lower median gestational age at delivery in that period follows the timing of the procedure rather than a change in outcome. No fetal, neonatal, or perinatal death occurred in either period. Adverse neonatal morbidity is defined as in Table 3. GA, gestational age; NICU, neonatal intensive care unit.

Supplementary Table S14. Number of observations available for each variable reported in Tables 1 to 3

Variable	Late<br>(24–32+0 weeks)<br>(N)	Early<br>(<24 weeks)<br>(N)
Pregnancy-level variables (Tables 1 and 2)	N = 18	N = 25
Maternal age	18	25
Nulliparity	18	25
Pre-pregnancy body mass index	18	25
IVF conception	18	25
History of preterm birth	18	25
Chorionicity	18	25
Gestational age at cerclage	18	25
Indication for cerclage	18	25
Cervical length at cerclage	18	24
Cervical dilatation or bulging	18	25
Irregular uterine contractions	18	25
Vaginal bleeding	18	25
C-reactive protein at cerclage	18	21
White blood cell count at cerclage	18	25
Vaginal progesterone use	18	25
Antibiotic use	18	25
Gestational age at delivery	18	25
Cerclage-to-delivery interval	18	25
Delivery within 7, 14 and 28 days	18	25
PPROM within 48 hours	18	25
Clinical chorioamnionitis	18	25
Preterm birth <37, <34, <32 and <28 weeks	18	25

Neonatal-level variables (Table 3)	N = 36	N = 46
Birth weight	36	46
Neonatal death	36	46
NICU admission	36	46
Respiratory distress syndrome	36	46
Intraventricular hemorrhage	36	46
Bronchopulmonary dysplasia	36	46
Necrotizing enterocolitis	36	46
Hypoxic-ischemic encephalopathy	36	46
Neonatal sepsis	36	46
Adverse neonatal morbidity	36	46

Data are the number of pregnancies, or of liveborn neonates, for which the variable was recorded. Two variables were incomplete and both gaps fell in the early group. Cervical length at cerclage was not measured in one pregnancy in which cerclage was history-indicated at 14+4 weeks, and C-reactive protein at cerclage was not requested in four pregnancies, three of them history-indicated procedures placed at 14 to 15 weeks. A blood count was drawn in all four of those pregnancies. No pregnancy and no neonate was excluded because of a missing value, and every summary in Tables 1 to 3 rests on all available observations. IVF, in vitro fertilization; NICU, neonatal intensive care unit; PPROM, preterm premature rupture of membranes.
